# Changes in Posttraumatic Brain Edema in Craniectomy-Selective Brain Hypothermia Model Are Associated With Modulation of Aquaporin-4 Level

**DOI:** 10.3389/fneur.2018.00799

**Published:** 2018-10-02

**Authors:** Jacek Szczygielski, Cosmin Glameanu, Andreas Müller, Markus Klotz, Christoph Sippl, Vanessa Hubertus, Karl-Herbert Schäfer, Angelika E. Mautes, Karsten Schwerdtfeger, Joachim Oertel

**Affiliations:** ^1^Department of Neurosurgery, Faculty of Medicine, Saarland University Medical Center, Saarland University, Homburg, Germany; ^2^Institute of Neuropathology, Faculty of Medicine, Saarland University Medical Center, Saarland University, Homburg, Germany; ^3^Faculty of Medicine, University of Rzeszów, Rzeszów, Poland; ^4^Department of Radiology, Faculty of Medicine, Saarland University Medical Center, Saarland University, Homburg, Germany; ^5^Working Group Enteric Nervous System (AGENS), University of Applied Sciences Kaiserslautern, Kaiserslautern, Germany; ^6^Department of Neurosurgery, Charité University Medicine, Berlin, Germany

**Keywords:** traumatic brain injury, decompressive craniectomy, brain edema, hypothermia, aquaporin-4

## Abstract

Both hypothermia and decompressive craniectomy have been considered as a treatment for traumatic brain injury. In previous experiments we established a murine model of decompressive craniectomy and we presented attenuated edema formation due to focal brain cooling. Since edema development is regulated via function of water channel proteins, our hypothesis was that the effects of decompressive craniectomy and of hypothermia are associated with a change in aquaporin-4 (AQP4) concentration. Male CD-1 mice were assigned into following groups (*n* = 5): sham, decompressive craniectomy, trauma, trauma followed by decompressive craniectomy and trauma + decompressive craniectomy followed by focal hypothermia. After 24 h, magnetic resonance imaging with volumetric evaluation of edema and contusion were performed, followed by ELISA analysis of AQP4 concentration in brain homogenates. Additional histopathological analysis of AQP4 immunoreactivity has been performed at more remote time point of 28d. Correlation analysis revealed a relationship between AQP4 level and both volume of edema (*r*^2^ = 0.45, *p* < 0.01, ^**^) and contusion (*r*^2^ = 0.41, *p* < 0.01, ^**^) 24 h after injury. Aggregated analysis of AQP4 level (mean ± SEM) presented increased AQP4 concentration in animals subjected to trauma and decompressive craniectomy (52.1 ± 5.2 pg/mL, *p* = 0.01; ^*^), but not to trauma, decompressive craniectomy and hypothermia (45.3 ± 3.6 pg/mL, *p* > 0.05; ns) as compared with animals subjected to decompressive craniectomy only (32.8 ± 2.4 pg/mL). However, semiquantitative histopathological analysis at remote time point revealed no significant difference in AQP4 immunoreactivity across the experimental groups. This suggests that AQP4 is involved in early stages of brain edema formation after surgical decompression. The protective effect of selective brain cooling may be related to change in AQP4 response after decompressive craniectomy. The therapeutic potential of this interaction should be further explored.

## Introduction

Traumatic brain injury (TBI) remains one of the main causes of death and disability in developed countries (1–3). What determines a patient's outcome following TBI is not only the degree of primary injury, occurring during trauma by mechanical force application to the head. As it was proven, the following series of pathophysiological changes known as secondary injury plays a crucial role in determining post traumatic recovery (4–6). As a consequence of secondary brain damage, edema, and a consequent raise of intracranial pressure (ICP) may develop (7). If this condition remains resistant to standard care, raised ICP may be the major contributing factor for the fatal outcome (8–13).

Among second-tier therapy options in neurotrauma, two methods recently evoked the researchers' particular interest. Firstly, decompressive craniectomy (i.e., partial surgical removal of skull bone) could be demonstrated as a method of efficiently relieving increased intracranial hypertension, reducing brain edema formation and improving neurological outcome after head trauma in several animal experiments (14–16). However, in clinical setting the beneficial effect is limited: One of two recent multicenter randomized controlled clinical trials on decompressive craniectomy (RescueICP) reported that surgical treatment decreased mortality after TBI, however at the cost of increased number of severely disabled patients up to 12 months after trauma (17, 18). More so, the previous of the randomized craniectomy trials (DECRA) suggested a deleterious impact of surgical decompression on neurologic outcome (17, 18). This conclusion could be supported by various experimental studies (including our own analyses), reporting increased structural damage and poorer functional recovery in animals treated by surgical decompression after head injury or subarachnoid hemorrhage (19–22).

**Figure 1:**
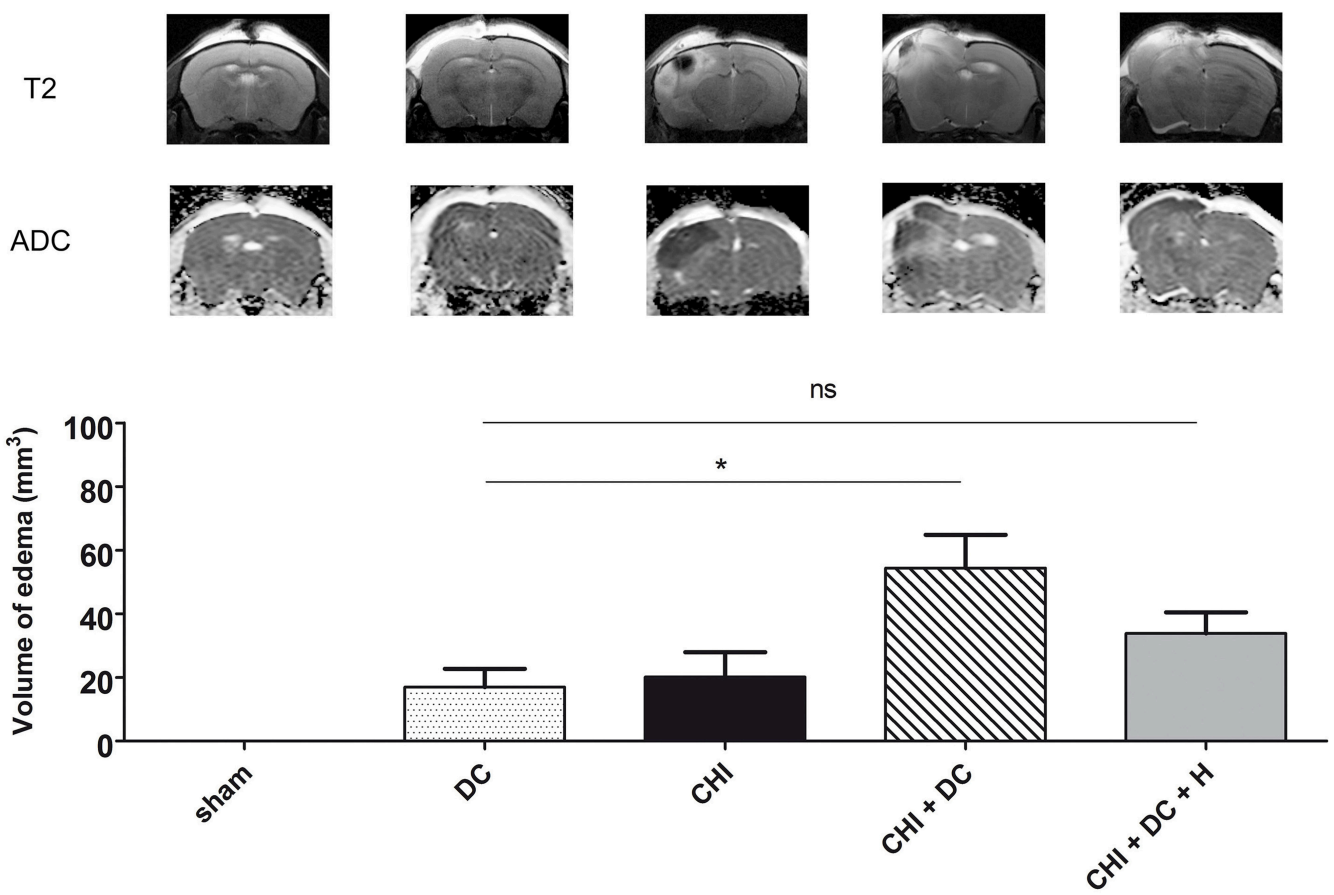
Demonstrating radiological sequelae (in particular brain edema) of trauma/surgery in experimental groups used for the current analysis. The upper panel displays the representative MRI scans obtained 24 h after trauma/sham treatment using a 9.4 Tesla scanner. There are apparent differences between the groups in pattern of brain edema (with CHI + DC group being most severely affected), as presented on T2-weighted images and on ADC maps. Below, the histogram represents quantitative analysis of ADC maps in regard to volume of brain edema. The volumetric data obtained here were used as one of the variables in correlation analysis in current study (*, *p* < 0.05; ns, *p* > 0.05). ADC, apparent diffusion coefficient; CHI, closed head injury; DC, decompressive craniectomy; H, hypothermia; MRI, magnetic resonance imaging. Adapted from Szczygielski et al. (39); © Mary Ann Liebert, Inc., New Rochelle, NY. Adapted with permission.

The other of these mentioned second-tier therapies, cerebral hypothermia, was hoped to be an efficient method to attenuate secondary brain damage mediated by its ICP-reducing and neuroprotective properties, evident both in animal experiments (23–27) and single-center clinical settings (24, 28–32). Unfortunately, hope for the efficacy of systemic hypothermia in improving patients' long term outcome was refuted in large multicenter clinical trials (33–36), mostly due to severe systemic side effects including electrolyte derangement, coagulopathy, and infectious complications (34, 37, 38). Therefore, whole body cooling has been abandoned as a standard therapy for TBI. To achieve reported neuroprotective effects of hypothermia without risk of previously mentioned systemic side effects, selective, or focal brain cooling got into focus. Some previous studies (conducted also by our group, see Figure 1) were able to report a limitation of brain edema formation due to focal application of hypothermia (20, 39–45).

Obviously, reducing of brain edema is the main target in management of raised intracranial pressure. Canonical work published by Klatzo et al. distinguishes between vasogenic brain edema, resulting from damage to the blood-brain-barrier with subsequent extracellular water accumulation and between cytotoxic brain edema, where water excess gathers in the intracellular compartment of neurones and astrocytes (46). Later on, Marmarou and associates refined these definitions, pointing out that energy depletion, necessary for active maintenance of ion-water homeostasis is the main pathomechanism in cytotoxic brain swelling (7, 47–51).

This dichotomy is represented also in radiological studies, visualizing both brain edema types by implementing magnetic resonance imaging (MRI). For example, estimation of brain edema character may be provided *in vivo* by measuring of water particles diffusion in tissue and demonstrated as difference of intensity in apparent diffusion coefficient maps (ADC). Using this technique, a heterogeneous (both vasogenic and cytotoxic) character of posttraumatic edema has been documented (52, 53). Importantly, the proportion of both edema types changes within the posttraumatic course, with cytotoxic edema (demonstrated as hypointense ADC areas) being the predominant form of swelling during acute phase (54–56). This MRI-based observation was made also in experiments based on closed head injury (CHI) model (57–60) used in our laboratory (22).

Posttraumatic brain edema formation in its both forms is governed by many molecular interplayers. One of these, aquaporin-4 (AQP4), deserves particular attention. AQP-4 is a water channel protein that is present on astroglial foot processes, near to cerebral capillaries or CSF spaces (61). Numerous studies report a crucial role of AQP4 in development and resolution of brain edema of any origin, e.g., of ischemic (62–64), hemorrhagic (65–68), infectious (69, 70), and traumatic one (71–75). During the time course of brain edema formation following primary injury, the task of AQP4 changes significantly and depends strongly on underlying edema subtype (vasogenic vs. cytotoxic), differing by injury character (71, 74–77). For the analysis of the AQP4 role in cerebral edema development, above cited distinction between two forms of cerebral edema (cytotoxic vs. vasogenic) is of great importance: The role of AQP4 differs diametrically between vasogenic and cytotoxic brain swelling (74, 78, 79), with AQP4 being usually increased in brain injury models demonstrating mostly vasogenic edema type (77, 80, 81). Furthermore, the most solid body of evidence is provided by experiments using AQP4 knockout mice. In ischemic stroke models, where mainly cytotoxic brain edema is represented, AQP4-deficient animals presented with reduced edema formation and improved functional outcome, both in models creating permanent and transient ischemia (62–64). Thus, an AQP4-mediated deleterious effect on blood-brain-barrier water permeability is indicated and a protective mechanism to reduce increase of cytotoxic edema formation trough AQP4-downregulation can be suggested. In contrast, in vasogenic edema, the role of AQP4 channels seems to be beneficial by facilitating the reabsorption of excessive fluid and thus the clearance of brain edema. Accordingly, animals lacking AQP4 presented with a greater amount of brain edema compared to wild-type littermates in model of central nervous system bacterial infection (69, 70) as well as in brain tumor model and cold brain lesion model (82), both being characterized by predominantly vasogenic brain edema.

Numerous pharmacological interventions targeted toward the reduction of posttraumatic edema formation exert their effect by AQP4 modulation (83–88). Importantly, impact on AQP4 expression / level could also be reported by experimental groups using decompressive craniectomy (16) or hypothermia (89, 90) as solitary treatment modes.

In a series of previous experiments, we elaborated a decompressive craniectomy mouse model based on the well-established paradigm of closed head injury (CHI) (22, 91, 92). Using this model, we were also able to successfully implement a combined treatment, composed of surgical decompression and subsequent focal cooling of the contused area. Accordingly, deleterious sequelae of combined trauma—decompressive craniectomy treatment were less prominent, if selective deep cooling of injury epicenter has been performed (20, 39). However, the molecular background of these phenomena remained unclear. Thus, for the purpose of current study we hypothesized, that in our model, the structural changes (in particular brain edema) and biochemical changes (AQP4 level) share the same pattern across the experimental groups. We also presumed, that AQP4 level correlates with extent of brain edema. In order to explore this hypothesis, we initiated a biochemical analysis of the brain tissue obtained in previously conducted experiments in order to show whether the reported detrimental impact of decompressive craniectomy as well as alleviating effect of selective brain hypothermia are associated with the influence on aquaporin-4 expression. Our second aim is to analyze the correlation between biochemical sequelae (AQP-4 level) and radiological features (edema/contusion) of TBI.

## Methods

### Animals and trauma model

All animal experiments were performed with approval by the local ethical board (28/2006, Saarland Ethical Commission), in line with the laws for animal protection, including Directive 2010/63/EU and by following all institutional and national guidelines for the care and use of laboratory animals.

Male wild-type, CD-1 mice of 9–12 weeks of age, without previous surgical or drug treatment, weighting 35.49 ± 0.59 g were acquired from the Charles River Germany GmbH & Co and kept in local Animal Facility of Institute for Clinical and Experimental Surgery.

Before starting the experimental procedure, mice were randomly assigned in one of the following experimental groups: 1. sham-operated (sham); 2. closed head injury alone (CHI); 3. decompressive craniectomy alone (DC); 4. CHI followed by DC at 1 h post-TBI (CHI+DC); 5. CHI+DC and selective hypothermia maintained for 1 h (CHI+DC+H) (*n* = 5 animals from each group, suitable for analysis as described further).

For the surgical part of the experiment, isoflurane anesthesia protocol has been established basing on recommendations of several Animal Welfare Agencies (93) and under assent of local Representative of Animal Welfare Board, Saarland University. According to protocol, spontaneously breathing mice were kept under general anesthesia by isoflurane inhalation (Forane®, Baxter, administered via Isoflurane Vapor® 19.1 device, Dräger; initial dose 3% in 97% O_2_, maintenance 0.8–1.5%, in 99.2–98.5% O_2_).

For groups 2, 4, and 5, experimental TBI was induced using a weight drop device [adapted from Chen et al. (91)]. Briefly, the animals were placed on a heating pad with an additional heat lamp used if necessary. Target core and head temperatures were measured by a rectal probe and a needle temperature probe placed in the right temporal muscle, respectively and maintained at 37 ± 0.5°C during the whole experiment. Following a midline longitudinal head skin incision the skull was exposed, the head was placed manually on the base of the weight drop device (Laboratory Tools Workshop, Department of Pharmacology, School of Pharmacy, The Hebrew University of Jerusalem, Israel). A 75 g weight was dropped from the height of 30 cm on a silicone cone resting on the exposed skull, resulting in focal brain injury to the left hemisphere. For groups 1 and 3 (sham and decompressive craniectomy alone), the same procedure was performed without weight dropping. In the CHI+DC and CHI+DC+H groups, an unilateral DC was performed 1 h after trauma as described previously (22). In brief, a bone flap was created in the parietal and temporal bone using a dental drill. The temporal bone was then removed down to the skull base and dura was opened above occipital lobe using microscissors and microforceps. Subsequently, skin was closed using 6-0 polypropylene sutures (Premilene®, Aesculap AG).

In DC group, the same procedure was performed on non-traumatized brain/skull 1 h following sham injury.

In hypothermia group (CHI+DC+H), additional selective brain cooling was applied using a carbon dioxide-driven cryosurgery device as described in detail previously (39). For selective, controlled cooling of the traumatized area, a modified cryosurgery apparatus was used. In hypothermia group a 3 mm cooling probe with thermocoupling (Erbokryo AE, ERBE Elektromedizin GmbH) was placed on the skin covering the decompressed area and chilled to 4°C. Utmost care was taken in order to avoid compression of the underlying brain in the process of cooling. After reaching target temperature, consecutive cooling was maintained for 1 h.

After 3 h, and after assuring the adequate whole body temperature (≥37°C) rectal and temporal temperature probes were removed and anesthesia was withdrawn. Animals were put back into their cages and allowed to recover including passive rewarming in an environment with controlled room temperature without additional heating devices.

### Magnetic resonance imaging

Twenty-four hours after CHI or sham treatment, animals (*n* = 5/group) were enrolled in imaging experiments. For MRI, anesthesia was induced after placing the animals in an airtight box, by applying a 3.0/97.0% mixture of isoflurane and O_2_ to the spontaneously breathing animals. Anesthesia was maintained by application of a 2.0/97.5% to 0.8/99.2% mixture of isoflurane and O_2_ via a nose cone integrated into the animal frame. Respiration rates were recorded via a pneumatic cushion (Graseby infant respiration sensor, Smith Medical Germany, Grasbrunn, Germany), while cardiac rates were collected via electrodes for neonatal humans (3M Red Dot 2269T neonatal monitoring electrode, 3M Germany, Düsseldorf, Germany), both with a dedicated animal monitoring system with integrated external computer and special software (PC-SAM32, SA Instruments Inc., Stony Brook, NY, USA). Temperature was maintained at 37°C by placing the animals on a special tray with an integrated heating system.

MR images were acquired using a system developed for rodent imaging, with a static magnetic field strength of 9.4 T (Bruker BioSpec Avance III 9.4/20 with ParaVision 5.1 operating software), equipped with an actively shielded gradient (G_max_ 675 mT/m, Gradient Rise Time 114.8 μs). An actively detuned single channel volume coil with an inner diameter of 70 mm, a maximum peak pulse power of 1,000 W and a maximum single pulse energy of 5 Ws served as transmitter (in transmit-only mode). For receiving MRI signals, an actively decoupled pretuned phased array surface coil with 2x2 elements designed for imaging of the mouse brain was placed over the skull and centered over the brain midline. After placing the animal in the isocenter of the magnet, a FLASH localizer sequence was performed (Field of View 3.84 × 3.84 cm^2^, Matrix Size = 256 × 256, Slice Thickness 1 mm, Interslice Distance 0.5 mm, TR/TE = 100/20 ms, Number of Excitations = 2, Duration 25 s 600 ms) generating a set of five subsequent slices in axial, sagittal, and coronal orientation. The symmetry axis of the brain was identified, evaluating the position of the inner and outer parts of the ear and various lobes of the cerebellum and cerebrum. A 3D FISP sequence (Field of View 1.76 × 1.50 × 1.73 cm^3^, Matrix Size = 236 × 200 × 23, resulting Slice Thickness 0.75 mm, Interslice Distance 0.0 mm, TR/TE = 8.0/4.0 ms, Number of Excitations = 3, Duration 1 m 25 s 423 ms) in axial orientation was then used to verify correct positioning with symmetric imaging of the brain, and slice geometry data was loaded into standard T1 and T2 weighted MRI sequences and an Echo Planar Imaging technique.

T1 weighted imaging for morphological analysis and planning of T2 weighted and DWI experiments was performed with a Multi Slice Multi Echo technique (Field of View 1.76 × 1.50 cm^2^, Matrix Size = 234 × 200, Slice Thickness 0.75 mm, Interslice Distance 0.0 mm, Number of Slices = 23, TR/TE = 1,000/10 ms, Number of Excitations = 4, Duration 13 m 20 s), generating a set of images covering the whole brain.

Matching axial images for identification and quantification of possible hemorrhage were acquired with a Turbo Spin Echo (TSE) sequence (Field of View 1.76 × 1.50 cm^2^, Matrix Size = 234 × 200, Slice Thickness 0.75 mm, Interslice Distance 0.0 mm, Number of Slices = 23, TR/TE = 2,500/30 ms, Number of Excitations = 5, Duration 5 m 12 s).

For accurate quantification of brain tissue inflicted by edema, axial diffusion-weighted echo planar imaging was performed with the following parameters: Field of View 1.92 × 1.92 cm^2^, Matrix Size = 192 × 192, Slice Thickness 0.75 mm, Interslice Distance 0.0 mm, Number of Slices = 7, TR/TE = 2,000/18.2 ms, Number of Excitations = 1, Duration 48 s, B Values of 6.45 s mm^−2^ and 786.74, 789.19, and 789.19 s mm^−2^ in sagittal, axial, and coronal direction.

**Figure 2:**
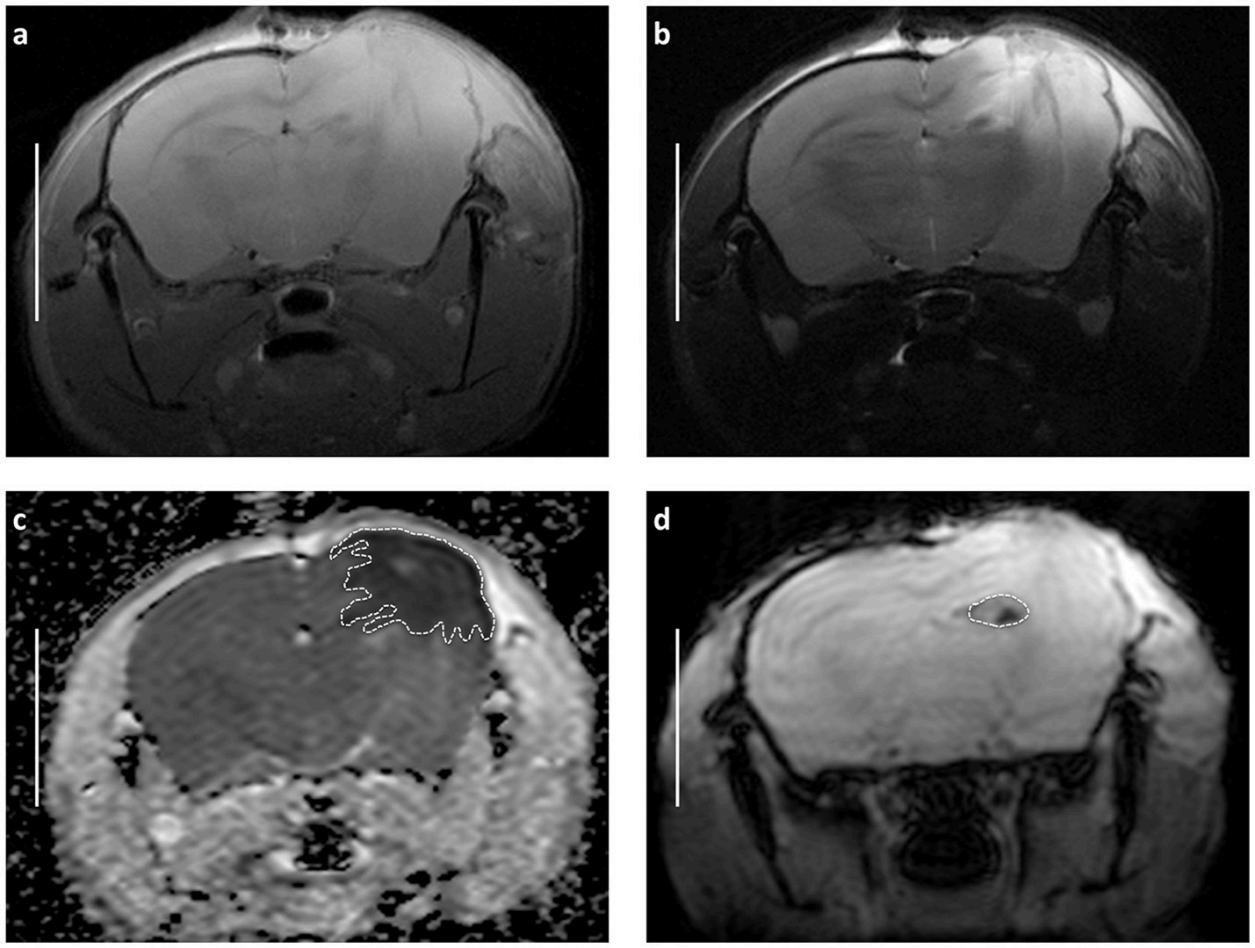
Demonstrating main radiological features of decompressive craniectomy in closed head injury model and approach for volumetric assessment of MRI images basing on representative scans obtained 24 h after trauma and craniectomy (CHI + DC group). **(a)** T1-weighted image **(b)** T2-weighted image **(c)** Appartent diffusion coefficient (ADC) weighted map. The dashed line defines the area of edema as marked manually by an independent observer. The area measurement on series of slices was followed by calculating of edema volume by defined slice thickness. Note the size and character of swollen region (marked hypointensive area underlying the craniectomy window). These features suggest domination of cytotoxic brain edema involving vast cortical areas after surgical decompression. **(d)** The same MRI layer presented in T-RARE sequence. Here, the area of hemorrhage is outlined by dashed line. Again, the calculation of contusion volume is performed based on the areas of contusion on single slides and on the slide thickness. Bar = 5 mm.

Edema and hemorrhage were identified in ADC maps calculated from the DWI data and in TSE images, respectively. Matching Regions of Interest (ROI) were manually created with the Paravision 5.1 ROI tool (example presented in Figures 2a–d). Resulting size measurements (in pixels and mm^2^) were exported via a specially adapted macro, and the total volume of the different lesions was calculated from the areas on the single maps and the thickness of the scan slices after importing the data into Microsoft Excel 2003® for Windows XP® and thereafter into GraphPad Prism® 5.0 for further analysis (see Statistics).

### Biochemical analysis (ELISA)

Twenty-four hours after trauma or sham injury, animals were sacrificed using *in situ* freezing with liquid nitrogen while under inhalative anesthesia (sublethal concentration of isoflurane 3.5–4% in O_2_). Snap frozen brains were dissected from surrounding tissue and brain stem and cerebellum were discarded. Thereafter cerebrum was dissected and the region of interest (ROI) was separated from the remaining brain tissue (ROI was defined as the brain tissue located −0.1/+ 0.1 cm from the point of maximal injury (virtual in trauma groups or hypothetical in experimental groups without trauma), seen in the brain coronal slice presenting CA1 and CA3 hippocampal areas). For this purpose, frozen brain specimens were cut in the coronal plane using gross section setting of cryotome (Leica, working temperature: −20°C, slice thickness 50 μm). Between four gross sections, one regular thin slice (12 μm) was obtained, stained with haematoxylin-eosin and analyzed under light microscope (Olympus, magnification 40x and 100x) for comparison with stereotactic mouse brain atlas (94) in order to confirm the proper cutting plane and ensure anatomical reference for ROI. The gross sections were diligently collected, and parts representing ipsi- and contralateral hemisphere were separated. In that manner, four separate samples from each animal brain (*n* = 5/group), as referring to the site and distance from epicenter of (hypothetical) injury (ipsi- vs. contralateral x ROI vs. remnant tissue) were obtained. Thereafter, specimens were stored at −80°C until final processing. For analysis of AQP-4 level in brain tissue, ELISA method was used. Frozen samples were lyophilized overnight. Dried tissue was then homogenized (FastPrep24, MP Biomedical) and resuspended in 1:10 PBS (DPBS, Dulbecco). Protein concentration was measured (Quant-It Assay, LifeTechnologies) and concentrations adjusted to 20 mg/mL. Aquaporin-4 concentrations were measured in a 10-fold dilution with a mouse Aquaporin 4-ELISA Kit (Hoezel Diagnostika, Germany; Reference number 90582Mu) according to the manufacturer's protocol. All samples were measured in duplicate on a Genios (Tecan, Germany) plate reader at 450 nm and concentrations were calculated with the Magellan software (Tecan, Germany).

### Histological analysis (immunohistochemistry)

In order to gain more detailed information about spatial distribution and time course of AQP4 expression, additional subset of animals has been used and histological analysis of AQP4 immunoreactivity was performed 28d after initial treatment (see also Supplementary Materials and Methods).

### Statistical analysis

Values of AQP4 concentration were recorded as pg/mL (of origin homogenizate). For each experimental animal, data set of four values has been obtained (AQP4 concentration in: 1. ipsilateral ROI; 2. ipsilateral remote area; 3. contralateral ROI; 4. contralateral remote area). Aggregating of data matched according to anatomical descriptors (lateralization: ipsi- vs. contralateral; longitudinal proximity: ROI vs. remote area of the brain) was performed and assessed supplementary to the analysis of the distinct data sets (values matched according to both anatomical descriptors). To avoid a pseudo replication bias, the aggregated data have been averaged, so that the one animal contributed only one value to the analysis. Both separate and aggregated parameters were expressed as mean ± SEM for each experimental group. For assuring the Gaussian distribution character of sampled data, Shapiro-Wilk test retrieving *p*-value as validation of normality for single group was performed. For data sets with confirmed Gaussian distribution of values, one-way ANOVA was implemented, otherwise Kruskal-Wallis test followed by Dunn‘s multiple comparison test was used for single analysis step.

To analyze the correlation between size of structural damage (volume of edema or volume of contusion) and between biochemical marker (tissue level of AQP4), the matching data from the single animals were analyzed (zero-value outliners of sham groups being excluded) according to Pearson correlation coefficient and a subsequent linear regression analysis method was performed.

For all parts of assessment (analysis of variance, correlation analysis), significance was set at *p* < 0.05 and statistical software GraphPad Prism version 5.00 for Windows, GraphPad Software, San Diego California USA, www.graphpad.com as well as IBM SPSS Statistics for Windows, Version 22.0 IBM Corp. Released 2013. Armonk, NY: IBM Corp. was used. In order to verify the validity of the analysis and to assess the risk of type II error in small cohort study, G^*^Power software, Version 3.1.9.2. was used for *post-hoc* assessment of the statistic power for both ANOVA and correlation analysis (95, 96).

## Results

### AQP4 concentration

**Figure 3:**
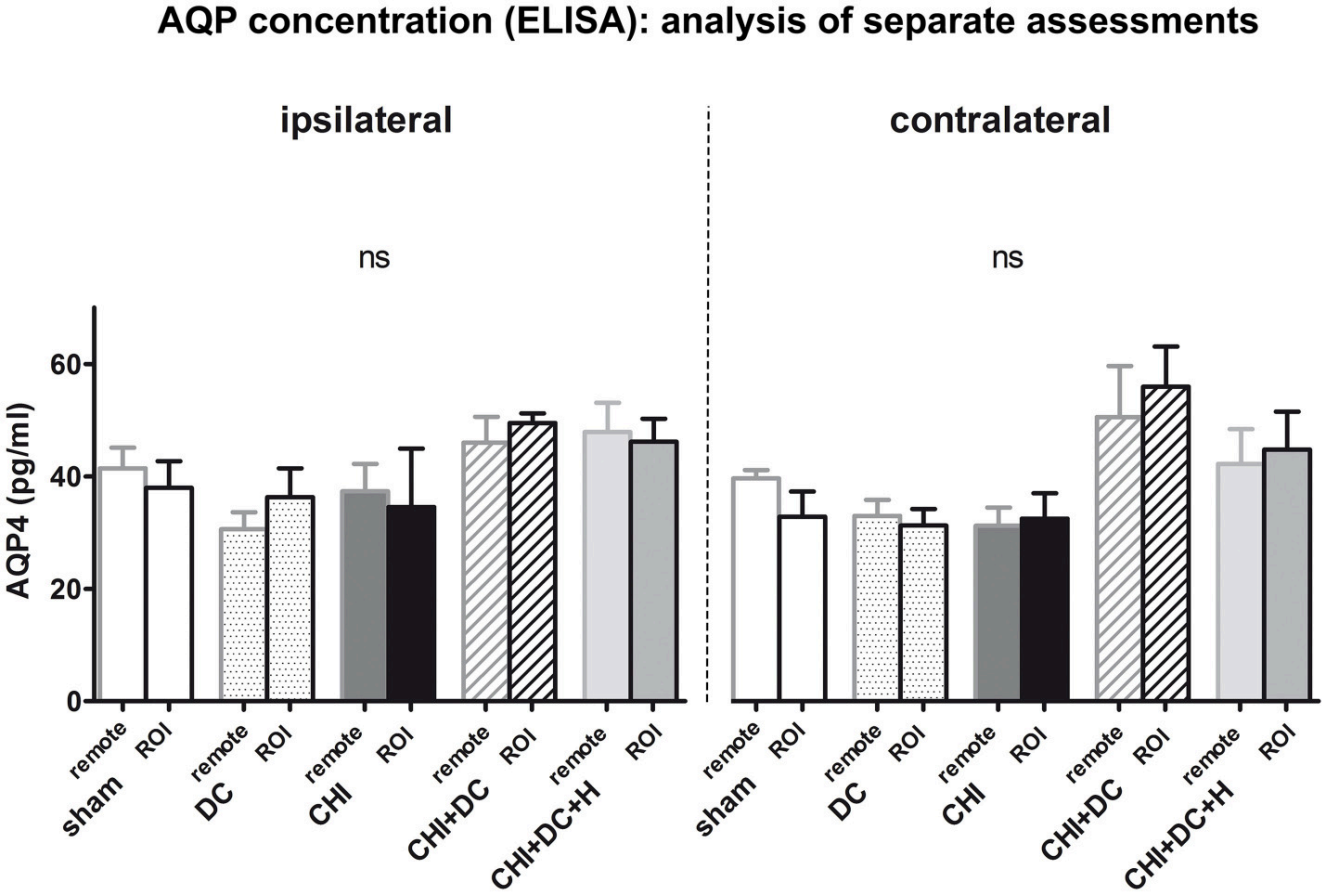
Histogram showing concentration of AQP4 24 h after trauma/sham injury according to ELISA assessment. The graph represents separate analysis of data i.e., the values are discriminated as to the localization of the sample (ROI vs. remote parts of the brain and ipsi- vs. contralateral hemisphere). Both the response to injury (AQP4 level change) and the differences between single treatment groups seem to be more prominent in contralateral hemisphere (containing more viable tissue), although according to the ANOVA analysis, no statistically significant differences can be registered (ns, *p* > 0.05 for comparison of groups, matched in distance or laterality in regard to injury epicenter). CHI, closed head injury; DC, decompressive craniectomy; H, hypothermia; ROI, region of interest.

Analysis of AQP4 concentration revealed no significant difference between experimental groups / areas, if concentrations were calculated separately for ROI vs. remaining tissue in ipsi- vs. contralateral hemisphere (Figure 3).

**Figure 4:**
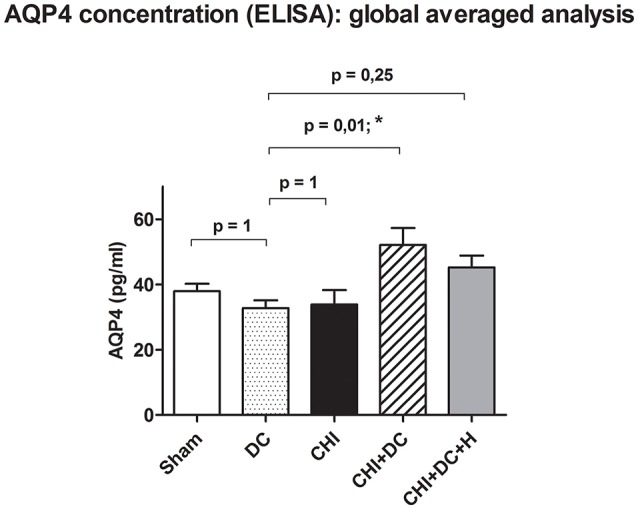
Histogram showing concentration of AQP4 24 h after trauma/sham injury according to ELISA assessment. The graph represents analysis of global pooled data i.e., the values obtained separately at the level of ROI (ipsi- and contralateral) as well as outside the ROI (ipsi- and contralateral) have been pooled and averaged. According to the ANOVA analysis, the global AQP4 level was significantly higher in the animals subjected to trauma and craniectomy as compared to craniectomy-only group, serving as reference group (CHI+DC vs. DC, *p* = 0.01, *). This effect vanished, when additional hypothermia has been applied (CHI+DC+H vs. DC, ns, *p* > 0.05). CHI, closed head injury; DC, decompressive craniectomy; H, hypothermia; ROI, region of interest.

However, analysis of aggregated values of AQP4 concentration (mean ± SEM) presented a statistically significant increase in AQP4 level in animals subjected to decompressive craniectomy after trauma compared to decompressive craniectomy alone (DC: 32.8 ± 2.4 pg /mL vs. CHI + DC: 52.1 ± 5.2 pg/mL, *p* = 0.01; ^*^). Notably, if additional hypothermia after surgical decompression was applied, this effect could not be documented (DC vs. CHI + DC + H: 45.3 ± 5.6 pg/mL, *p* > 0.05; ns) (Figure 4), although direct comparison between the groups CHI + DC vs. CHI + DC + H presented no statistical significance (*p* > 0.05; ns); (*post-hoc* statistical power analysis for ANOVA: power of 0.98 by effect size *f* = 1.01).

**Figure 5:**
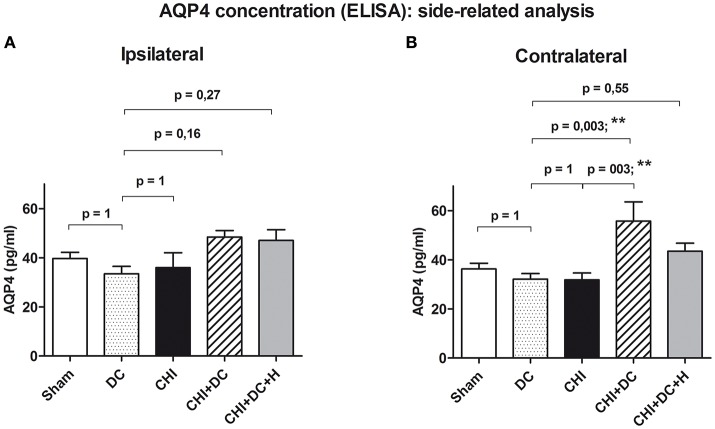
Histogram presenting concentration of AQP4 24 h after trauma/sham injury according to ELISA assessment. This part of analysis involves the data pooled and averaged separately for the contralateral hemisphere (at the level of ROI plus outside the ROI) and for the ipsilateral one (again, at the level of ROI plus outside the ROI). **(A)** According to ANOVA, the differences between AQP4 level values, averaged for ipsilateral hemispheres were not significantly different between the treatment groups. **(B)** Analogous analysis in contralateral hemispheres revealed significant raise in AQP4 concentration in animals subjected to trauma and decompressive craniectomy as compared to non-traumatized reference group as well as to trauma-only group (CHI+DC vs. DC, *p* < 0.01, **; CHI+DC vs. CHI, *p* < 0.01, **). Similar to global pooled analysis, this effect could not be seen in group where hypothermia was added to the treatment (CHI+DC+H vs. DC, *p* > 0.05, ns). CHI, closed head injury; DC, decompressive craniectomy; H, hypothermia; ROI, region of interest.

More detailed analysis revealed, that the effect of decompressive craniectomy, increasing AQP4 level in global aggregated calculation, resulted from an increase of AQP4 level in non-traumatized hemispheres, since the difference between decompressive craniectomy animals (DC) and CHI+DC group as well as between trauma animals and decompressive craniectomy group was statistically significant in aggregated analysis of AQP4 concentration parameters in contralateral but not in ipsilateral hemispheres (for ipsi: DC: 33.4 ± 3.1 pg/mL, vs. CHI + DC: 48.5 ± 2.6 pg/mL, *p* > 0.05; ns; CHI: 35.9 ± 6.1 pg/mL vs. CHI + DC, *p* > 0.05; ns); (*post-hoc* statistical power analysis for ANOVA: power of 0.70 by effect size f = 0.65); for contra: DC: 32.1 ± 2.3 pg/mL vs. CHI + DC: 55.8 ± 7.8 pg/mL, *p* < 0.01; ^**^; CHI: 31.8 ± 2.8 pg/mL vs. CHI + DC *p* < 0.01; ^**^); (*post-hoc* statistical power analysis for ANOVA: power of 0.97 by effect size f = 0.97); (Figures 5A,B).

### Correlation of AQP4 concentration with radiological sequelae of TBI

The results of volumetric analysis of edema and contusion [as described previously by our group (39) and demonstrated by Figure 1] provided one of the variables for subsequent correlation analysis. As second variable, results of ELISA AQP4 assessment were adapted.

**Figure 6:**
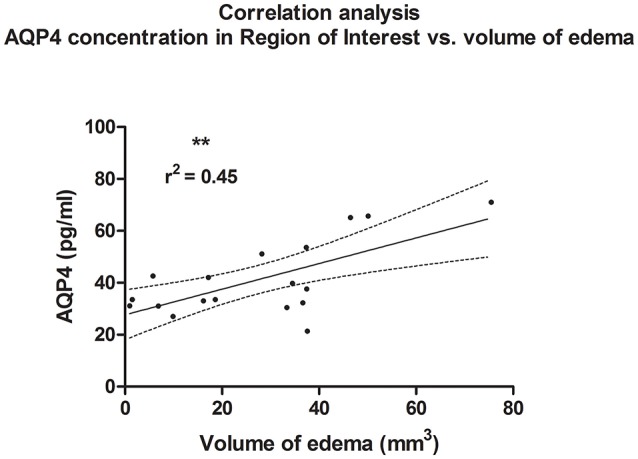
Histogram presenting analysis of correlation between volume of edema 24 h after trauma/sham injury and AQP concentration, assessed in contralateral hemisphere at the level of injury (ROI contralateral). There was a correlation between increased volume of brain edema and elevated concentration of AQP4 according to ELISA (*r*^2^ = 0.45; *p* = 0.003; **).

**Figure 7:**
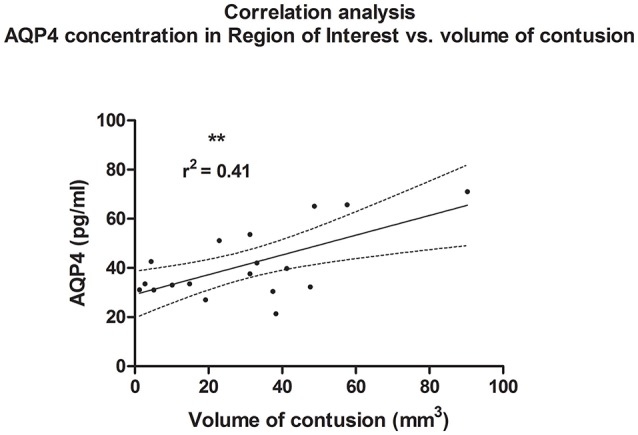
Histogram presenting analysis of correlation between volume of contusion 24 h after trauma/sham injury and AQP concentration, assessed in contralateral hemisphere at the level of injury (ROI contralateral). Similar to analysis of edema size, a correlation between increased volume of contusion and elevated concentration of AQP4 according to ELISA (r^2^ = 0.41; *p* = 0.004; **) could be demonstrated.

A linear correlation analysis demonstrated a correlation between edema volume measured in ipsilateral hemisphere and between concentration of AQP4 assessed in the ROI contralateral to the injury site (*r*^2^ = 0.45; *p* = 0.002; ^**^); (*post-hoc* statistical power analysis for correlation: power of 0.98 by effect size ρ = 0.67); (Figure 6). Also, contusion volume was correlated with the AQP4 level in the corresponding region (ROI) of the contralateral hemisphere (*r*^2^ = 0.41; *p* = 0.004; ^**^); (*post-hoc* statistical power analysis for correlation: power of 0.97 by effect size ρ = 0.64); (Figure 7).

### Histological analysis

Qualitative analysis of histopathological material yielded observation similar to previous anatomical description of AQP4 immunoreactivity in mice (97, 98) without any statistical difference between groups (see also Supplementary Results and Figure S1).

## Discussion

In our previous experiments on the effects of decompressive craniectomy and hypothermia in a murine CHI model, we were able to demonstrate an increase of brain edema formation and neurological impairment due to the combined effect of mechanical trauma and surgical decompression. We also observed a mitigation of this deleterious effect of surgical decompression by consequent focal cooling of the traumatized brain area via the created craniectomy window (20, 22, 39). Our current results demonstrate the potential molecular background of these phenomena: In the same set of experimental animals these processes are associated with the change in AQP4 level affecting remote brain areas rather than trauma epicenter at the analyzed time point 24 h posttrauma.

The main result of our current analysis is the increase of AQP4 concentration in animals subjected to both trauma and decompressive craniectomy. That is completely opposing the observation of Tomura et al. who reported significant increase of AQP4 expression level affecting at 48 h only animals not subjected to surgical decompression, while the use of decompressive craniectomy reduced both brain water content and AQP4 protein expression in rat model of fluid percussion injury (FPI) (16). To explain this discrepancy, the differences between animal TBI models with regards to their pathophysiological characteristics should be discussed in the light of general principles of brain edema formation (as presented in Introduction). First, FPI model is characterized by diffuse injury pattern (reaching brain stem or even cerebellum) (99–102). In contrast, trauma-decompression model used in our study represents a rather focal injury, where trauma epicenter (including contusional changes) evokes pathophysiological response of the surrounding tissue (22, 39). Second, in FPI model, presence of vasogenic brain edema already at early stages of the posttraumatic course have been described (99–102). Opposite, in CHI the initial posttraumatic edema is predominantly cytotoxic, as presented in previous reports (57–60) as well as in former radiological assessments performed by our group (22). As learned from the AQP4 knockout animal experiments (69, 70), the molecular response by AQP4 expression in posttraumatic brain edema depends on the predominant edema form (vasogenic vs. cytotoxic) and this rule is to be extrapolated into traumatic brain injury models. An overview of selected studies on AQP4 implementing different models of experimental TBI is demonstrated in Table 1.

**Table 1:** Summary of reported AQP4-related changes in different animal models of TBI.

**Study**	**Animal**	**Trauma model**	**Additional intervention**	**AQP4 changes**	**Method of assessment**
Ke et al. (103)	Rat	Weight drop	None	↓	RT-PCR AQP4- mRNA
Kiening et al. (104)	Rat	CCI	None	↓	WB
Zhao et al. (105)	Rat	CCI	TBI + Sulforaphane (vs. TBI)	↓↑	IF
Taya et al. (71)	Rat	CCI	None	↑	WB
Tomura et al. (16)	Rat	FPI	TBI + Decompressive craniectomy (vs. TBI)	↑↓	IC
Quintard et al. (106)		FPI	TBI + MLC901 (vs. TBI)	↔ (7d) ↑ (7d)	IF, WB
Fukuda et al., (107)	Rat	CCI	None	↑	IF
Gatto et al. (84)	Rat	CCI	TBI + rhEPO (vs. TBI)	↑↓	WB
Zhang et al. (75)	Rat	CCI	None	↑ (>24 h)	WB
Blixt et al. (108)	Rat	CCI/Weight drop	None	↓	IF/WB
Jin et al. (109)	Rat	FPI	TBI + Levetiracetam (vs. TBI)	↑↓	WB, RT-PCR AQP-mRNA /cDNA
Zhang et al. (86)	Rat	CCI	TBI + Astaxanthin (vs. TBI)	↑↓	RT-PCR AQP-mRNA, WB
Blixt et al. (87)	Rat	CCI/Weight drop	TBI + Erythropoetin (vs. TBI)	↓↑	IF
Szczygielski et al. (this paper)	Mouse	CHI	TBI + Decompressive craniectomy (vs. TBI)	↔↑	ELISA

*Description of animal models: CCI, controlled cortical impact; CHI, closed head injury; FPI, fluid percussion injury. Description of assessment methods: cDNA, complementary deoxyribonucleic acid; ELISA, enzyme-linked immunosorbent assay; IC, immunohistochemistry; IF, immunofluorescence; mRNA, messenger ribonucleic acid; RT-PCR, real-time polymerase chain reaction; WB, Western blot*.

In studies using controlled cortical impact model (characterized by predominant cytotoxic edema formation) (48, 110, 111) decreased posttraumatic AQP4 level in brain tissue has been revealed (104, 105, 108) although Taya and associates could report an increase in AQP4 expression in the early stages after CCI (71). Moreover, interference with AQP4 expression (107) or function (84, 86, 87, 105) by pharmacological intervention resulted in decreased posttraumatic brain edema formation and improved neurological outcome in studies using CCI trauma model. In contrast, in fluid percussion injury model, where vasogenic mechanism of swelling plays an important role (56, 53) due to BBB breakdown (112–114), trauma resulted in a surge of AQP4 concentration (16, 106) and/or in increased AQP4 gene expression (109).

In the light of this data on relationship between AQP4 function and form of brain edema, a certain mismatch in our set of results needs to be admitted: The previous description of CHI model, as well as our own radiological results suggests that cytotoxic edema prevails in our experimental setting. At the same time, the observed changes in AQP4 level follow the pattern characteristic of vasogenic edema type, at least in the trauma + decompressive craniectomy group, as characterized by raise in AQP4 concentration. In detail: According to primary description, CHI model is characterized by the domination of cytotoxic edema, at least in the early phase (1–24 h after injury). This has been presented in previous MRI studies performed in experiments using CHI (57–60). Also our own observation, showing predominance of hypointense areas of ADC maps (to be identified as regions of cytotoxic edema) is sound with the previous evidence (22). On the other hand, the observed pattern of AQP4 changes (especially correlation analysis) suggests participation of vasogenic edema in early sequence of events following surgical decompression. In particular, previous publications describing stroke-related brain swelling, were able to report a negative correlation of edema volume and AQP4 level or expression in the phase of cytotoxic edema formation, while the development of vasogenic edema was closely linked with AQP4 increase, the latter seen also in our analysis (77, 80, 81). Hypothetically, a raise in AQP4 concentration in vasogenic edema formation may represent the attempt of functional brain tissue to counteract the rapid increase of extracellular fluid (115). This role of AQP4 is confirmed by previous experiments analyzing inflammatory edema (115) which shares some characteristics with perifocal edema accompanying intracerebral hematoma (115, 116). Notably, in the presented trauma-decompression model, a substantial amount of tissue injury results from hemorrhagic transformation of the traumatized cortex with secondary perifocal edema formation (22, 39). A similar involvement of AQP4 has also been documented in edema formation surrounding the clot in animal models of intracerebral hemorrhage (65, 116–118) with spontaneous and traumatic hematomas sharing main features of perifocal brain swelling (119, 120). These previous observations match our current analysis documenting a close correlation between AQP4 level and the size of contusional changes.

Certainly, the method of radiological analysis (MRI ADC mapping) provides only restricted information about edema type. However, not only the character of AQP4 reactive changes allows us to speculate that vasogenic edema was involved in pathophysiology of brain edema after decompressive craniectomy in our model. One mechanistic link is the character of pressure changes usually following surgical decompression. If the raised intracranial pressure is relieved by surgical opening of the cranial vault, the pressure gradient between blood vessels and brain parenchyma rapidly changes (21), possibly promoting vasogenic edema formation due to hydrostatic driving force (121, 122). Also changes in cerebral perfusion associated with deranged cerebrovascular autoregulation as seen in post-craniectomy patients (123–125) may lead to hyperemia and thus, to the enhanced vasogenic brain edema formation (19, 46). Notably hyperperfusion could be well correlated with the degree of brain edema amount measured on CT scans in clinical settings (126). Possibly this aspect provides a valid explanation for above mentioned discrepancy between our results and those provided by Tomura et al. In our model (other than in the study of Tomura and associates), extensive brain edema development after decompression was not restricted by meticulous control of blood pressure and subsequent hydrostatic gradient (16), leading to early, massive brain swelling (demonstrated by increased water content and external brain herniation, as previously reported) (20, 22). Possibly this uncontrolled brain edema development seen already 6 h posttrauma (20) lead to impaired neurological recovery and increased neuronal loss (22). These observations vary from reports of other groups reporting neuroprotective effects of experimental surgical decompression after TBI (14, 15, 127). Significant differences of the TBI model used (diffuse vs. focal trauma pattern) as well as differing injury severity may be quoted as reasons for the diverse conclusions of animal studies on decompressive craniectomy.

Another important conclusion from our results is that the influence on AQP4 concentration caused by treatment modality differs according to the traumatized brain region [albeit one cannot exclude a bias, relying on difference in ANOVA power, as calculated *post-hoc* for analysis of contra- and ipsilateral hemispheres (128, 129)]. The laterality of posttraumatic changes in AQP4-level deserves particular attention. The different impact of head injury on AQP expression ipsi- and contralateral to trauma site has been analyzed in previous animal studies. In a rat TBI weight drop model, reduced AQP4 mRNA expression has been reported in the ipsilateral but not in the contralateral hemisphere (103). Also following controlled cortical impact in the rat, intensity and time course of AQP4 concentration differed between ipsi- and contralateral hemispheres and a decrease of AQP level in the lesion core parallel with an increase in the penumbra zone was described (104). Furthermore, the problem of inhomogenous AQP4 expression across the traumatized brain has been closely approached in several studies. A clear difference between trauma epicenter (AQP4 reduction) and penumbra zone (AQP4 increase) could have been stated (103, 105, 130). A decrease in AQP4 level, that has been attributed to the necrotic transformation of the core of contused area (105, 131) fits well to our observation: As reported previously, a vast area of ipsilateral cortex in our model was affected by necrotic changes and hemorrhagic contusion, most prominent in the trauma + craniectomy group (22). The following upregulation of AQP4 level may be blunted by the severe loss of AQP4 expressing cells, which is more abundant in the ipsilateral hemisphere, while the contralateral viable tissue, remote to the injury epicenter is able to execute this compensatory mechanism in less restricted way. This hypothesis is further supported by studies targeted strictly on the contralateral brain tissue, since early (<24 h) AQP4 overexpression in a rat model of severe TBI could be seen in the cortex contralateral to injury site (132). Also Zhang and associates describe the difference between ipsi- and contralateral hemispheres considering molecular response and emphasize delayed dynamics of AQP4 peak in areas contralateral to injury (75).

Finally, we would like to discuss the influence of focal brain cooling on AQP4 expression pattern. According to our results, we suggest that hypothermia potentially ameliorates the posttraumatic edema course, which reflects in reactive changes of AQP expression. Several analyzes previously investigated the impact of hypothermia on AQP4 expression / function. Results of cell culture studies were not conclusive: Fujita et al. reported a reduction of AQP4 expression in cultured astrocytes subjected to temporary hypoxia, followed by a secondary raise in AQP4 mRNA expression when subjected to mild hypothermia. In the normothermic condition, induced AQP4 depletion was sustained (133). In an astrocyte culture setting of Lo Pizzo et al. hypothermia led to a reduced AQP4 level (133). In contrast, Salman et al. report an increased presence of membrane AQP4 channels in cortical astrocytic cultures under hypothermic condition without a change in global AQP4 protein expression (134). However, cell culture models do not necessarily recollect intricacy and dynamics of AQP4-related posttraumatic changes in the mammalian brain. Therefore, several groups analyzed the influence of hypothermia on AQP4 expression/function *in vivo*, using rodent models of hypoxia / ischemia. In these models, AQP4 level increase caused by ischemia (135) or ischemia-reperfusion injury (136) could be attenuated by reducing cerebral temperature, albeit this effect seemed to be strongly dependent on rewarming conditions (137). Results, most relevant for our current analysis could have been provided by Gao et al. (138). In this work, focal brain cooling reduced both AQP4 expression and concentration following a stereotactic injection of thrombin, simulating deep intracerebral hematoma in rat. This observation suits well our results, considering the fact that the protective effect of hypothermia is mediated by a limitation of contusional changes (39, 42) and that contusion volume correlates well with AQP4 level in our current analysis. Thus, selective brain hypothermia may reduce not only the size of contusional hematoma but may also diminish its negative impact on surrounding tissue. Several mechanisms of action need to be discussed. First, direct impact of reduced temperature on the AQP4 gene expression in affected cells may be postulated (138). This however does not explain the remote influence of focal hypothermia. This effect relies rather on modulation of inflammatory mechanisms postulated by Kurisu et al. (136): It has been proven, that both localized hematoma and focal injury may trigger the inflammatory reaction and blood brain barrier (BBB) disturbance even in distant parts of the brain (139–141). Thus, the remote effect of injury may be limited, if the course of focal events (inflammation or BBB-breakdown) is arrested by hypothermia (142). Nevertheless, this hypothesis implies that the observed changes of AQP4 level need to be interpreted as only secondary indicator of injury severity. Regardless the underlying mechanism, our study is (according to our best knowledge) the first report to describe the effect of focal cooling of the traumatic lesion core on AQP4 level in an experimental setting.

Another advantage of our study we would like to highlight is the animal species used. We deliberately focused on a murine model of closed head injury model as the basis for our craniectomy experiments, even if performing this procedure in small rodents requires experimenters' particular manual dexterity. This effort is gratified by the future possibility to convey this experimental design into genetically modified animal models, usually being mouse breed. This step could be of particular value for the further development of AQP4 targeted treatment strategies, as has been stressed by Yao et al. In this work, drugs or maneuvers applied following TBI in order to alter AQP4 expression have been judged critically as since AQP4 knockout mice subjected to CCI displayed only a mild improvement of neurological function and lesion volume compared to wildtype littermates as well as only a transient beneficial effect of AQP4 knockout on brain water content (74). Again, the above-mentioned heterogeneous character of cerebral edema formation following TBI as well as the kinetics of cytotoxic and vasogenic edema contribution over the posttraumatic course have to be considered. A premature extrapolation of experimental results into clinical context may be counter-productive, if the predominantly underlying edema subtype in the considered posttraumatic phase is not respected (74). Thus, the role of AQP4 in the different stages of posttraumatic course should be analyzed in animal models tightly resembling clinical setting. The advantage of our experimental paradigm is the sequence of moderate to severe TBI followed by early craniectomy performed on non-trephined skull, similar to clinical scenario, including trauma severity warranting indication for surgical decompression (22). By performing decompression on an intact skull, the potentially confounding disadvantage of skull trepanation prior to injury can be avoided (143–146). In order to respect the above-mentioned “true sham” effect in experiments requiring skull trepanation, we deliberately chose the decompression-without-trauma (DC) group as primary reference group for statistical evaluation. In our previous studies, we were able to validate this strategy (22, 39), again showing most profound changes in the group subjected to both trauma and subsequent craniectomy.

Certainly, our study is not free of several drawbacks. First, the analysis is limited to the time point 24 h postinjury, as predetermined by the setup of our previous experiments (22, 39). According to previous reports, focused on the radiological time course of brain edema development, the early course of posttraumatic changes is characterized by cytotoxic edema type, while vasogenic swelling peaks not before 3 days after primary insult (52, 102, 115, 116). According to this, current preliminary analysis possibly does not display the full diversity of brain edema formation under different combinations of treatment. Certainly, we have tried to compensate this gap by presenting the preliminary data from long-term analysis, implementing AQP4 immunohistochemistry staining (see Supplementary Data). However, the results reported here did not reveal any significant difference between treatment groups. This observation is in concordance with Fukuda et al. who demonstrated mitigated brain edema formation and reduced AQP4 expression (due to administration of small interfering RNA) 3 days after trauma, but without any change in AQP4 immunoreactivity as assessed 60 days post injury (107). Second, since the current results are based on offshoot analysis of brain tissue material obtained previously, the choice of method for AQP4 analysis was limited. Certainly, immunostaining method or microdissection of the anatomical structures would provide more detailed information about spatial AQP4 expression. However, even using our microtome-based, rough method for separation of different areas of the traumatized brain we were able to demonstrate inhomogenity in molecular AQP4-based response at single time point of edema build-up phase. Finally, as predefined by the setup of the source experiment, current biochemical analysis could be performed in very limited number of animals. This is the possible cause of inhomogeneous statistical power across single analysis steps (ANOVA for ipsilateral AQP4 level with power <0.8, while in other reported parts of analysis the risk of the type II error was quite low with the *post-hoc* power value of 0.97–0.98). The low number of animals resulted also probably in differences between single treatment groups becoming apparent first after aggregating of AQP4 concentration values. This, however, is in concordance with the previous observations, reporting no significant effect of trauma on AQP4 expression up to 48 h post injury (16). Also Yao et al. analyzing the impact of AQP4 knockout after TBI stated the influence of AQP4 depletion on posttraumatic course to be minimal (74). This leads to expect only a scarce difference in AQP4 level, which justifies form of analysis (aggregation of the single data). Nevertheless, we overcame the temptation of plainly multiplying the data set and analyzing repeated measures as independent values. Instead, we chose to average the aggregated data, which is a simple, yet effective method to reduce the flaw resulting from pseudoreplication of individual records (147).

In conclusion, the increase of brain edema formation following decompressive craniectomy in a murine model of severe CHI is accompanied by an increase in AQP4 level. This elevation seems to be reactive and most probably represents an attempt to resolve extracellular water, possibly resulting from a disturbed hydrostatic gradient following mechanical decompression. Due to the focal injury character caused by the weight drop model, the molecular changes differ across the various regions of traumatized brain. Nevertheless, the extent of this posttraumatic response seems to be governed by the core lesion volume. Due to our results, selective application of focal hypothermia at the injury epicenter is associated with less prominent AQP4 response even in remote areas of the brain. Certainly, this effect may be secondary. However, basing on our preliminary animal experiments we recommend further analysis of this phenomenon in order to explore the therapeutic potential of i.e., pharmacological influence on AQP4 expression/function as treatment strategy supplementary to decompressive craniectomy and/or hypothermia.

In spite of these promising data, therapeutic implications of the AQP4-modulating effect on cerebral swelling should at this stage be taken with caution, since our results were provided using a limited number of experimental animals. More importantly, cerebral edema formation occurring after trauma and subsequent decompressive craniectomy (both in animal models and in clinical settings) is apparently represented by a dynamic mixture of cytotoxic and vasogenic brain edema with a beneficial or deleterious property of AQP4 strongly depending on ratio of these two constituents.

## Author contributions

JS, K-HS, AEM, KS, and JO contributed conception and design of the study. JS, CG, CS, and VH conducted the animal experiments. JS, CG, MK, CS, and K-HS performed the biochemical analysis. AM and VH performed the radiological assessment. JS, AM, MK, and K-HS performed the statistical analysis. JS wrote the first draft of the manuscript. AM and VH wrote sections of the manuscript. All authors contributed to manuscript revision, read, and approved the submitted version.

### Conflict of interest statement

The authors declare that the research was conducted in the absence of any commercial or financial relationships that could be construed as a potential conflict of interest.

## Abbreviations

AQP4: aquaporin 4
ADC: apparent diffusion coefficient
BBB: blood brain barrier
CCI: controlled cortical impact
cDNA: complementary deoxyribonucleic acid
CHI: closed head injury
DC: decompressive craniectomy
DWI: diffusion weighted image
ELISA: enzyme-linked immunosorbent assay
FPI: fluid percussion injury
H: hypothermia
IC: immunohistochemistry
ICP: intracranial pressure
ICU: intensive care unit
IF: immunofluorescence
IR: immunoreactivity
MRI: magnetic resonance imaging
mRNA: messenger ribonucleic acid
RARE: rapid acquisition with refocused echoes
RNA: ribonucleic acid
RT-PCR: real-time polymerase chain reaction
TBI: traumatic brain injury
TSE: turbo spin echo
WB: Western blot.
