# Supplementary material for: Changes in Posttraumatic Brain Edema in Craniectomy-Selective Brain Hypothermia Model Are Associated With Modulation of Aquaporin-4 Level
Source: Front Neurol. 2018 Oct 2;9:799. doi: 10.3389/fneur.2018.00799 (PMC6176780; doi:10.3389/fneur.2018.00799)
Supplement: Supplementary file 1 [file Data_Sheet_1.docx]

**Changes in Posttraumatic Brain**

**Edema in Craniectomy-Selective**

**Brain Hypothermia Model Are**

**Associated With Modulation of**

**Aquaporin-4 Level**

*Jacek Szczygielski, Cosmin Glameanu, Andreas Müller, Markus Klotz,*

*Christoph Sippl, Vanessa Hubertus, Karl-Herbert Schäfer, Angelika E. Mautes,*

SUPPLEMENTARY DATA *Karsten Schwerdtfeger and Joachim Oertel*

**Supplementary Materials and Methods (Immunohistochemistry)**

For the purpose of more detailed spatial analysis of AQP4 expression and distribution, additional subset of animals (n = 8 each group) has been subjected to the treatment as described in the main text. In order to demonstrate impact of treatment at more remote time points, the animals were sacrificed 28d after initial treatment using transcardial perfusion with buffered formaldehyde solution; the brains were removed and fixed in the same solution for 7 days. The brains were paraffin embedded and serial coronal sections of the brains (5 µm) were made, presenting region of interest (ROI) i.e. the coronal slices displaying hippocampal areas CA1 and CA3 as assessed using the stereotactic mouse brain atlas ([Paxinos and Franklin, 2001](#_ENREF_92)) (see References main text). Thereafter, the slices were immunostained with anti-AQP4 antibody according to following procedure: Coronal sections were deparaffinized and rehydrated. Antigen retrieval was performed by boiling sections in 10 mM citrate buffer (pH 6.0) for 20 min. After cooling for 20 min and washing with TRIS puffer, sections were incubated with 0.3% H_2_O_2_ in methanol for 20 min and washed with TRIS, thereafter intrinsic binding sites were blocked using 5% goat serum plus biotin/avidin blocking kit (Vector Laboratories; SP-2001), and incubated with rabbit polyclonal AQP4 antibody with mouse reactivity (Santa Cruz Biotechnology; sc-20812, dilution 1:100) at 4 °C overnight. After washing with TRIS puffer, sections were incubated with biotinylated goat anti-rabbit antibody (Vectastain Elite ABC HRP Kit (Peroxidase Rabbit IgG) Vector Laboratories; PK-6101) at room temperature for 60 min and thereafter washed with TRIS puffer for 10 min. Following, sections were incubated with an avidin-biotin-peroxidase system (Vectastain Elite ABC HRP Kit (Peroxidase Rabbit IgG) Vector Laboratories; PK-6101) for 20 min and washed with TRIS puffer for 10 min. Thereafter slices were stained using chromogen kit (Vector VIP Peroxidase HRP Substrate Kit; Vector Laboratories; SK-4600) for 20 min, washed with distilled water for 3 min and thereafter counterstained with hematoxylin.

The coronal section underwent qualitative analysis by an independent observer blinded to treatment of the animal, utilizing a light microscope (Olympus, x40/x100/x200). Additionally, a semiquantitative analysis was performed: under 200 x magnification, the areas CA1 and underlying stratum radiatum of hippocampus (with well-contrasted, less abundant AQP4 immunoreactivity, as previously reported ([Hubbard et al., 2015](#_ENREF_47)) (see References main text) were identified and count of immunoreactive cells was assessed. Here, the immunoreactivity four-grade score was arbitrary used according to the following description: 1 - single cells, 2 - slight increase of immunoreactity, 3- moderate increase of immunoreactity and 4-intensive increase of immunoreactivity.

Values of histopathological semiquantitative analysis were recorded (as ordinal data) and expressed as mean ± SEM for each experimental group for further analysis (Kruskal-Wallis test followed by Dunn`s multiple comparison test), using the statistical software as described in the main text.

**Supplementary Results**

Qualitative analysis of histopathological material yielded observation similar to previous anatomical description of AQP4 immunoreactivity in mice ([Hsu et al., 2011](#_ENREF_46); [Hubbard et al., 2015](#_ENREF_47)) (see References main text): Here, a diffuse, granular and bush-like staining of astrocyte network could be demonstrated in all groups of animals, differing slightly as to the intensity of immunoreactivity between sham and traumatized / surgically treated groups.

However, semiquantitive analysis did not reveal any significant difference in number of the AQP4 immunoreactive cells across different treatment groups (p > 0.05, ns for all compaired pairs; however, by relative low post hoc power value of 0.61 by effect size f = 0.46) (Fig. S1).


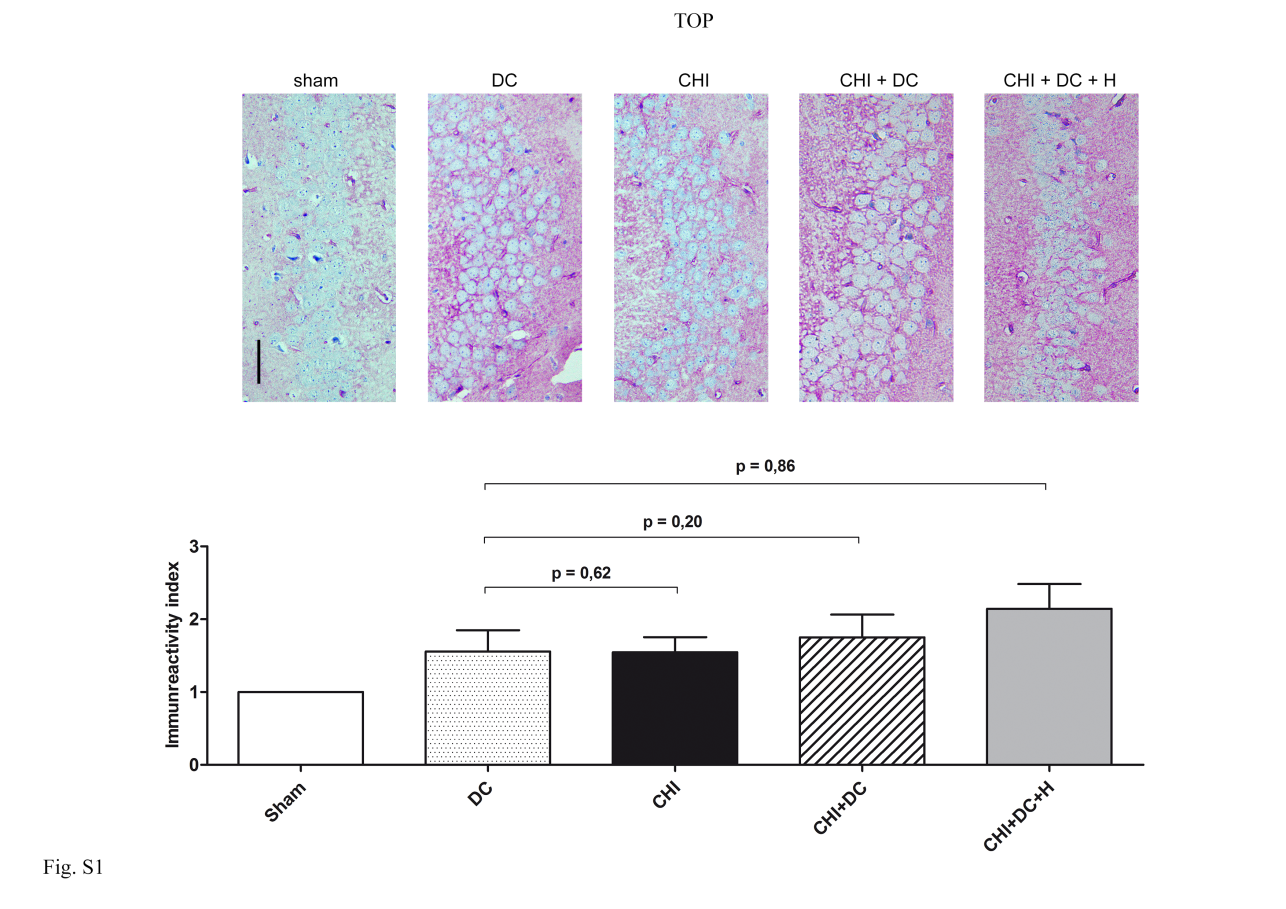


Fig. S1: Demonstrating late changes in AQP4 distribution (28d post trauma) in subset of animals subjected to histopathological analysis (for full size image see separate supplementary file).

The upper panel displays photomicrographs of coronal paraffin sections immunostained with anti-AQP4 primary antibodies. The least intense immunoreactivity is presented in naïve (sham treated animals). In groups subjected to surgery or /and trauma, a distinct pattern of bush-like networks of AQP4-positive astrocytes could be demonstrated in stratum radiatum, permeating also the cellular layer (stratum pyramidale) of CA1 region. Magnification 200x, scale bar: 50 µm.

Below, the histogram represents semiquantitative analysis of AQP4 immunoreactivity expressed as IR-index attributed by the independent observer. According to the Kruskal-Wallis analysis, no statistically significant differences can be registered (ns, p > 0.05 for comparison of all pairs of groups).

CHI = closed head injury, DC = decompressive craniectomy, H = hypothermia, IR = immunoreactivity
